# Measuring the Impact of Genetic and Environmental Risk and Protective Factors on Speech, Language, and Communication Development-Evidence from Australia

**DOI:** 10.3390/ijerph18084112

**Published:** 2021-04-13

**Authors:** Paula Cronin, Stephen Goodall

**Affiliations:** Centre of Health Economics, Research and Evaluation (CHERE), Faculty of Business, University of Technology Sydney, Ultimo 2007, Australia; stephen.goodall@chere.uts.edu.au

**Keywords:** language, speech, infants, childhood, risk factors, protective factors, environment, genetics, development

## Abstract

Speech and language acquisition is one of the key development indicators of optimal literacy development in infancy and early childhood. Over the last decade there has been increasing interest in the development of theoretical frameworks which underpin the underlying complexity of a child’s language developmental landscapes. This longitudinal study aims to measure the impact of genetic and environmental risk and protective factors on speech, language, and communication development (SLCN) among 5000 infants in Australia. Using robust panel fixed-effects models, the results demonstrate that there are clear and consistent effects of protective factors and SLCN associated with the infant’s family [coefficient (SD) = 0.153, 95% standard error (SE) = 8.76], the in utero environment [coefficient (SD) = 0.055, standard error (SE) = 3.29] and early infant health [coefficient (SD) = 0.074, standard error (SE) = 5.28]. The impact of family and in utero health is dominant at aged 2 to 3 years (relative to 0 to 1 years) across the domains of language and communication and more dominant from birth to 1 years for speech acquisition. In contrast, the evidence for the impact of genetics on SLCN acquisition in infancy, is less clear. The evidence from this study can be used to inform intervention policies.

## 1. Introduction

The first 1000 days of life are recognized as a critical and sensitive period in the acquisition of speech and language, where the foundations of optimal literacy development are established. There are several theoretical frameworks which seek to explain the underlying complexity of a child’s developmental landscapes [1]. In the economics literature, the “Heckman theory” or theory of “human capital development” suggests that an individual’s rate of development is determined by an initial vector of “skills and abilities”, which is largely determined by a complex interaction of biological factors (genetic, in utero environment and early infant health status) [2,3,4,5,6,7] and social factors (cultural, familial, socio-economic, community) [8,9,10,11,12,13,14,15]. Low language is a significant early childhood development concern, which is viewed as a “health shock” to a child’s initial vector of “skills”. It affects 6 to 7% of children and up to 17% once social disadvantage is considered [16,17,18,19,20]. The literature shows that low language is associated with a range of short- and long-term consequences, and these consequences can persist into childhood and adulthood. These children are at risk of starting school developmentally lagging behind their peers and as a result are more likely to require academic support [21] have academic difficulties during their school years and/or have lower educational attainment [22,23]. Research also reveals that emotional difficulties [24] cognitive deficits [25,26] and social adversity [27], are more common in children with low language, which can further contribute to academic disengagement and/or lack of success [28] and ultimately higher rates of unemployment or being under employed [29]. The estimated cost of lost productivity associated with SLCN in Australia is $15.099 billion (USD 9.52b, €8.71b, £7,61b) over a lifetime [22]. Although much of the literature to date has focused on the consequences of low language, there is a recognized need to understand the mediating factors of language development [14]. We know from the literature that targeted speech pathology interventions have the potential to save up to A$3.69 billion (USD 1.96b, €1.79b, £1.56b) in lost productivity in Australia [22], but this does not consider the potential gains associated with focusing on broader interventions. The aim of this study is to measure the impact of environmental and genetic risk factors on speech language and communication acquisition in infants. Considering both the short- and long-term consequences of low language, it is, therefore, crucial to target future intervention strategies which optimize language acquisition in infants.

## 2. Materials and Methods

This longitudinal study was part of the Longitudinal Study of Australian Children (LSAC), whereby infants and parents were interviewed initially in the first year of the child’s life (for information on risk factors during pregnancy, and the health of the infant at birth) and then subsequently every 2 years, to measure the child’s ongoing health and development. Interviewers were trained by psychologists to directly assess young children’s development profile, according to screening guidelines. The present study used data from a nationally representative sample of infants in the birth cohort of LSAC. The LSAC used a two-stage sampling design, stratified by state and geographic area and clustered by postcode and then child. Some remote postcodes were excluded, due to the costs associated with data collection and the population estimates were adjusted accordingly. The sample was weighted for attrition. Full details of the LSAC study design have been previously published [30]. The study was approved the Centre for Health Economics Research and Evaluation’s (CHERE) program ethics approval: UTS HREC REF NO. ETH18-2507.

### 2.1. Study Setting and Population

#### Outcome Variable—Speech Language and Communication Acquisition

The LSAC birth cohort was the primary source of data in this study as it tracks a sample of approximately 5000 infants at birth through their first 1000 days or life (2 waves). The advantage of using these longitudinal data is that it allows the exploration of the speech and language acquisition and social communication, while controlling for a rich set of individual and family characteristics.

For the purposes of our analysis, we used the CATALISE criteria to define a continuous variable of language ability [31]. This variable is labelled speech language and communication needs (SLCN). Because of the number of variables that cover SLCN in the LSAC, we follow Kling, Liebman, and Katz (2007) and Cronin (2020) to estimate a summary standardized index that aggregates information over multiple measures, thereby improving statistical power [22,32] The summary index is the simple average across standardized z-score measures of each component. The z-score is calculated by subtracting the mean and dividing by the standard deviation and assumes a mean value of 0, with a standard deviation (95% confidence interval) of ±1. We convert each component of the index so that a higher score is a better outcome (e.g., convert “parental concern” to “no parental concern”). 

The SLCN index includes 11 components, across five measures: communication and symbolic behavior scales (CSBS) [33], parent evaluation of development status (PEDS) receptive and expressive language concern, concerns about speech and language development [30], communication skills scales and MacArthur-Bates Communicative development Inventories (MCDI) [34]. By aggregating information into a summary index, it allows us to combine information from multiple sources (parental report, standardized testing) across the three domains of speech, language, and communication [31].

The panel nature of these data considers both the timing (age of the child) and frequency (number of times the measures are reported) of the components, which allows us to capture the infant’s language acquisition trajectory. Results are reported as an overall SLCN index and the individual domains (speech, language, and communication) separately. It was not possible to define the individual domains as mutually exclusive as several of the components overlap domains. Consequently, components are categorized by their primary focus. E.g., parent reported concern of speech and understanding is categorized in the “speech” domain. Speech pathology is considered in each index to account for identification of difficulties in each of the three domains. These measures are summarized in Appendix A, Table A1.

### 2.2. Explanatory Variables of Interest

To account for the impact that these risk and protective factors have on an individual’s SLCN development, we included several measures reported in LSAC (individual, perinatal, birth, family, community and genetic risk factors) as covariates in the analysis (Appendix A, Table 1) Because of the number of variables that cover environmental and genetic risk factors in the LSAC, we follow a similar approach to defining the main explanatory variables of interest in terms of standardized indices, focusing on four main risk domains: (i) family, (ii) perinatal, (iii) birth, (iv) and genetics. We convert each component of the index so that a higher score is a better outcome (e.g., convert “risk factor” to “protective factor”).

### 2.3. Family

To account for the different aspects of language that are sensitive to social and language background we considered information on socio-demographic characteristics including: ethnicity, educational level, employment status, household size, financial stress, and hardship and monthly household income, which was obtained through parent self-report. In addition, positive parenting was captured in three variables: self-efficacy, warmth, and hostility. A “family risk index” was then calculated as the equal weighted average of the z-score of 11 variables: socio-economic position, equivalized household income, non-English-speaking background (yes/no), indigenous (yes/no), parents health status, receiver of single parent allowance (yes/no), hardship (higher score is less hardship), stressful life (number of events), mother’s depression (K6 depression score), mother education (Year 12 and above), parental self-efficacy, warmth, and hostility (reverse coded).

### 2.4. Perinatal

Information on perinatal risk factors was considered to capture the effects of the in utero environment on the infant’s growth and development [7]. These included maternal age (squared, reverse coded), presence of perinatal care (number of medical appts), pregnancy healthy behaviors (smoking and alcohol, reverse coded), presence of mental health issues (yes/no), high blood pressure (yes/no) and gestational diabetes (yes/no) and were reported in wave 1 when the infant was 0 to 1 years of age. A “perinatal risk index” was calculated as the equal weighted average of the z-score of nine variables: prescription medication during pregnancy, antidepressants during pregnancy, alcohol consumption, smoking, high blood pressure, gestational diabetes.

### 2.5. Birth

Information on birth outcomes, namely gestational age at birth, birth weight and length, extra medical care at birth, whether it was a single of multiple birth and time spent in hospital were extracted from parent self-reported records, when the infant was 0 to 1 years of age. Low birth weight (LBW) was defined as weight at birth of less than 2.5 kg [35]. Gestational age was defined as late (42 weeks and above), on time (37 to 42 weeks), somewhat early (33 to 36 weeks) and very early (32 weeks or less) A “birth risk index” was calculated as the equal weighted average of the z-score of nine variables: LBW, gestational age, infants head circumference, infant’s length, infant required intensive care, infant required ventilator, number of days in hospital and single or multiple birth.

### 2.6. Community

Variables relating to the infant’s surrounding community were considered to capture any wider neighborhood effects on language development, often categorized as socio-economic index for areas (SEIFA), which is based on the five-yearly Census [36]. A “community risk index” was calculated as the equal weighted average of the z-score of five variables: SEIFA index of economic resources, SEIFA index of relative socio-economic advantage and disadvantage, Year 12 completion, SEIFA index of education, and occupation and employment rates in the area of residence.

### 2.7. Genetics

There is evidence in the literature that genetic influences play a significant role in early communication development [2,3,5,9], with notable links between a child’s language and a family member with a history of language or literacy difficulties and/or other developmental impairments such as behavioral problems. The LSAC had limited information the parent’s medical conditions and health as a child. We relied on self-reported measures of long-term medical conditions (yes/no) of both biological parents, and the presence of grandparent’s mental health or alcohol issues, to calculate the multi-generational health and social risk. A “genetic risk index” was calculated as the equal weighted average of the z-score of nine variables: parents body mass index (BMI), parents long-term health conditions, grandparent’s mental health (yes/no) and alcohol issues (yes/no).

### 2.8. Identification of Additional Factors Related to SLCN

The analysis considered that speech and language development can be influenced by other co-morbidities such as neurodevelopmental disorders and can commonly co-exist with conditions such as cognitive and motor dysfunction [31,37]. These other impairments are defined by one variable, whether the infant has special health care needs. Individual demographics (gender), general health and attendance at childcare (number or hours) are considered to account for the different aspects of language and communication that are sensitive to health and background.

### 2.9. Final Sample for Analysis

From the original sample of 5107 infants, 9 were excluded as outliers in the distribution of SLCN. A sample of 5098 infants, across 2 waves were used in the analysis, from birth to 3 years of age. To control for missing observations, we use ad hoc imputation methods outlined by Hox (2002) [38].

### 2.10. Statistical Analysis

Panel random effects analysis was used to explore the relationship between the independent variables of interest and SLCN. Results for the main indexes (Family, Perinatal, Birth, Genetics) were presented as standard deviation (SD) and standard errors (SE) and the level of significance was set at *p* < 0.10 + *p* < 0.05 *, *p* < 0.01 **. Interactions with time were also considered. Data were analyzed using STATA Statistics version 14.1 software StataCorp LP, College Station, TX, USA.

## 3. Results

### 3.1. Characteristics of Infants and Families

Characteristics of the infants and families are shown in Table 1 Overall, the mean SLCN of infants was −0.04 (standardized z-score) (range: −4.7–1.54) Average gestational age was 39.2 weeks (range: 22–50 weeks). Most infants were single births (96.8%), 53.7% were males, and on average they had one older sibling and were in excellent health. Approximately one in six infants used intensive care after birth (14.13%), which is consistent with the national average (1 in 5), but is higher than international estimates (average 6%), and as such is likely to reflect the high rates of caesarean sections in Australia (30% for first time mothers as of 2018) [39]. A small proportion required a ventilator (1.96%) or were born with a low birth weight (2.71%). The average stay in hospital was between 5 and 6.04 days (range: 0–354 days) depending on private health insurance status. In terms of family demographics, most families had two parents (99.9%) caring for the child, with a household weekly income (equivalized) of $678 (range 0–$4647) and low levels of financial hardship (0.33 out of possible 6). In terms of maternal characteristics, the average age of the mother was 31.5 years (range: 15–63 years). Many displayed healthy behaviors during pregnancy, by avoiding some foods (48.05%), avoiding alcohol (average 0.07 standard drinks per day), and smoking (average 0.71 cigarettes per day). On average, the mothers attended between 5 and 6 prenatal care visits during their pregnancy and they generally reported good mental health (4.5 out of 6), with high levels of parenting efficacy (8.49 out of 10) and warmth (4.51 out of 5).

### 3.2. SLCN Results

Table 2 presents the results of the main regression model looking at the impact of risk indices on SLCN for infants aged 0 to 3 years of age, reported for the full SLCN index and for each of the three domains: speech, language, and communication, separately. Column 1 presents results for the full SLCN index (defined by the five components across three domains of speech, language, and communication). The effect of family risk factors on SLCN, as shown in column 1, is 0.153 and is statistically significant at the 1% level. The magnitude of the coefficient implies that increasing family protective factors one standard deviation improves SLCN by approximately 0.2 standard deviations. To put this into context, the average annual gains in SLCN between 0 and 3 years of age is 0.05 SD (depicted by age 2 to 3 years in Table 2). This means that the impact a family has on a child’s language development is close to three times the gains made by age alone. Similarly, there were large and positive effects of perinatal risk (0.055 **) and birth risk (0.074 **) on SLCN.

The remaining columns of Table 2 show the regressions for the individual components of the SLCN index (speech, language, and communication, separately). The results show that environmental protective factors, such as family, perinatal health and birth outcomes have the largest impact on *communication* development, (measured by speech pathology, CSBS social composite and communication skills scales) when compared to *speech* (measured by speech pathology, CSBS speech composite and parental concern about speech) and *language* (measured by speech pathology, CSBS symbolic composite, PEDS expressive and receptive language concern, MCDI vocabulary and grammar scales). In terms of specific risk indices, the coefficient for family protective factors on *communication* development (col 4) is the largest (0.241 **), followed by birth (0.10 **) and perinatal protective factors (0.095 **). In terms of the other SLCN domains, it appears that both family and birth factors play an important role in the development of *speech and language*, but the magnitude of the effects are not as high. The coefficient for family and birth protective factors on *speech* development is 0.081 ** and 0.092 **, respectively. The coefficient for family and birth protective factors on *language* development is 0.143 ** and 0.052 **, respectively. In terms of individual infant characteristics, male infants (−0.073 **) (compared to females), infants with special health care needs (−0.048 **), with older siblings (−0.050 **) and those with average and below health (−0.104 **) (compared to excellent health) have poorer language development. The gender differences in SLCN appears to be mediated by strong communication skills in girls.

### 3.3. Mediating Factors of SLCN

Table 3 attempts to disaggregate these risk indices to identify the important mediating factors of SLCN (reported as significant effects at 5% level). For ease of interpretation, results are re-converted to “risks (negative)” and “protective (positive)” factors. The results showed that the family effects observed in Table 2 are largely mediated through parenting warmth and self-efficacy. Notably, the effect of maternal warmth on an infant’s communication, as shown in column 4, is 0.129 **, which equates to more than twice the gains associated with age alone. Consistent positive effects of maternal warmth are also observed in both speech (0.064 **) and language (0.042 **) acquisition. Maternal self-efficacy also has a positive impact on communication (0.058 **), speech (0.046 **) and language (0.034 **). Interestingly, infants from a non-English-speaking background have higher speech (0.070 **) and communication (0.047 **) skills by aged 3, but their language is lagging behind their English-speaking peers (−0.035 **).

In terms of perinatal risk (row 3), the most important markers appear to be mother’s age (one-year increase in age is associated with −0.069 ** lower communication and −0.049 ** lower speech), and healthy pregnancy behaviors. Notably, excluding high-risk foods during pregnancy (0.012 +) and reduced smoking appears to have a consistently positive effect on SLCN (0.029 ** and 0.038 ** for communication and speech, respectively).

In terms of birth risk factors (row 4), LBW and gestational age appear to be the most important birth risk factors for SLCN development. Infants with LBW recorded 0.071(NS) lower SLCN, which was largely driven by lower language development (−0.089 * +), when compared to normal weight infants. Similarly, infants who were born somewhat early (33 to 37 weeks) or very early (32 weeks and below), reported −0.018 * and −0.179 ** lower SLCN respectively, when compared to infants born on time (37 to 42 weeks). In contrast to the consistent environmental effects observed, there is mixed evidence of the effects of genetics on early SLCN. The effects of multi-generational (fathers, maternal medical condition) risk have a small and inconsistent effect on language acquisition. There are significant differences by age and across the components of multi-generational risk.

### 3.4. Interactions with Age of Child (Time)

The final stage of the analysis is to consider the complexity of how key environmental influences interact over time, and with an individual’s genetic makeup. To test the importance of these interactions, we re-estimate the analysis, including interactions of the key risk indices with time or age of the child. The coefficient can be interpreted as the effect of one standard deviation increase of family index for the sample when they are aged at either 1 to 2 years or 2 to 3 years. The basis for this interaction is that if the age of the child does not matter, then the size of the re-specified coefficients should be consistent with the average (from Table 2) and remain statistically significant.

Table 4 shows differences in SLCN growth trajectories by age. Notably, the results showed for risk factors associated with the family environment (row 1), a steeper trajectory was observed at aged 2 to 3 years (relative to 0 to 1 years) across the domains of language and communication (increase from average of 0.153 ** (all ages) to 0.170 ** (col 3) and 0.274 ** (col 4), respectively). In contrast, the rate of speech acquisition was consistently steeper at 0 to 1 years of age (relative to 2 to 3 years) for all risk indices (col 2). Not unexpectedly, the impact of birth risk factors on SLCN was steepest immediately following birth (row 4), while the effects of perinatal health were more evenly distributed across both age groups (0 to 3 years of age) (row 3). The effects of community and genetic risk factors on SLCN are mixed, with some negative effects observed at Age 0 to 1 years (i.e., decreasing SLCN as community socio-economic status (SES)/genetic protective factors increase). However, this pattern is reversed at aged 2 to 3 years, although not statistically significant, which suggest that the community and genetic effects on language development are not linear. These patterns highlight the significant heterogeneity that exists in language development by age and within the different domains of SLCN.

### 3.5. SLCN Algorithm at 2 to 3 Years

Using the interaction model from Table 4, we estimate the overall level of speech language and communication skills for infants at 2 to 3 years of age (Table 5). The basis for this algorithm is to quantify the impact of multiple risk or protective factors over time. From this algorithm, an infant’s SLCN trajectory can be estimated, based on their level of perinatal, birth, and family risk indices plus the impact of any intervention. Estimates are interpreted as standard deviations (SLCN is a standardized score which assumes a mean SLCN at 2 to 3 years of 0 ± 2 standard deviations.), which can be translated into clinically important differences. For example, an intervention focused on improving the family environment (+1 SD) can serve to compensate for a high-risk perinatal (−1 SD) and birth period (−1 SD), resulting in a slightly above average SLCN (average SLCN = 0.040 SD) of 0.106 (row 23). In other words, with appropriate intervention, infants with a poor start to life can catch up in their speech and language development by 3 years of age. On the other hand, a negative family environment (−1 SD) can be more detrimental to a child’s language development, even with protective perinatal (+1 SD) and birth factors (+1 SD) in place (calculated SLCN of −0.026) (row 27).

Overall, the results from this study show that environmental protective factors play a vital role in the development of speech, language, and communication for infants up to 3 years of age. The interaction specifications highlighted that risk models should consider not only the number of risk factors, but the interaction effects over time.

## 4. Discussion

The aim of this study was to explore the impact of risk and protective environmental and genetic factors on speech language and communication acquisition for infants aged from 0 to 3 years of age. Using robust panel fixed-effects models, the results demonstrate that there are clear and consistent effects of protective factors associated with the infant’s family, the in utero environment, and early infant health. It is well documented in the literature that the family environment is an important predictor of children’s language and literacy [37], particularly family socio-economic status (SES) [40,41] and the home literacy environment (HLE) [15,42]. Interestingly, the results from our study indicated that positive parenting had a greater role to play in early language development than explicit measures of SES or educational poverty. Several studies have investigated the underlying mechanisms by which SES influences speech and language acquisition. Hoff (2003) found that differences in maternal speech was the key mediating variable in language development [10]. The authors found that mothers with high SES used more articulate and longer sentence structures, resulting in longer interactions with their children. This finding was consistent with the work of Marshall et al., in a study of early years practitioners, which highlighted that “it is the way you talk to children” which is the most important factor in speech and language development [12]. It is plausible that the measures of maternal warmth and self-efficacy, which focuses on warm encounters, activities while keeping the child calm, busy, and in a routine, is describing the mediating effects of SES on SLCN, through the HLE [10,15,43].

In addition, our results support Barker’s Hypothesis, which suggests that fetal and infant origins can influence the development of disease later in life [7]. For instance, LBW infants have a higher risk of developing illnesses, developing congenital abnormalities and cognitive deficits [44,45]. Our study indicates that both perinatal and birth risk may additionally influence the development of impairments associated with speech and language.

In contrast to the consistent effects observed for environmental risk factors, our study found limited evidence of the impact of genetics on SLCN acquisition in infancy. These findings are consistent with the literature which investigated longitudinal patterns of speech and language acquisition using studies of twins [4,13]. The authors found that shared environmental influences appear to be dominant in early language, with a smaller though significant role for genetic factors [4]. This pattern appears to reverse by middle childhood, whereby genetic makeup becomes more important. The emergence of genetic influences in an older cohort has been observed in other studies looking at the impact of low language in middle to late childhood, which found that early intervention and parental investment (treatment and high-quality schooling) are successful in improving academic outcomes [46].

The authors of the twins study also suggest that there is a useful distinction to be made, in terms of etiology, in the acquisition of language and speech skills. Differences in young children’s language skills, appear to be largely due to environmental influences, while differences in speech skills, appear to be mostly due to genetic effects. This contrasts with the results of our study, which found clear and consistent effects of family and birth risk factors on *speech* acquisition between birth and 1 years. It is possible that the genetic information available in the LSAC did not adequately capture the genetic components relevant to speech acquisition. The variable in LSAC were limited to multi-generational long-term medical conditions (including speech) but did not capture any childhood conditions of the parents or grandparents. This poor association is demonstrated in Table 3 whereby small and inconsistent effects of genetic risk with speech and language were observed. It is also feasible that some of the components of family (such as SEP), act as proxies for genetic links in the absence of robust alterative measures in LSAC. Further research is required to disentangle these effects.

The differences in etiology and longitudinal development patterns have implications for the value of intervention in early infancy. The dominance of environmental factors over genetics suggests that interventions targeting the specific domains of language and communication at this age should be broad in scope, focusing on the in utero and home environment. In contrast speech development interventions at this age, which may more dominantly influenced by genetics should have a targeted component, which specifically focuses on speech remediation [15].

Our study highlighted higher rates of speech and communication acquisition for infants from non-English-speaking backgrounds, when compared to English speakers. Chan and Silva (2015) investigated first- and second-language attrition in a second-language environment and found that home influences, transferable lexical growth, and cognitive maturity may lead to Language 2 learners accumulating vocabulary and understanding more rapidly. [47].

Finally, we found inconsistent effects of the influence on the community environment on emerging language, which is consistent with the literature which found that neighborhood was positively associated with the early child development domains of physical health and wellbeing and social competence, rather than language. As children get older, neighborhood and peer effects were positively associated with literacy development through children’s social-emotional development, and engagement in school [46,48].

### Limitations

Several limitations need to be considered in the present study. The first is that it is recognized in the literature that there is wide interindividual variation in language development in children under 5 years of age. Studies by Centre of Research Excellence in Child Language have shown that language development can accelerate, plateau or even go backwards within the space of a year [49]. These fluctuations make it very hard to accurately identify and predict which children will have sustained low language difficulties using single measures. The strength of our study is that it uses 11 different assessments of speech, language, and communication skills over 4 years (5 measures at 0 to 1 years and 6 measures in 2 to 3 years). However, our sample was subject to missing observations, notably MCDI (22%) and CSBS scales (12%) which means that a proportion of the sample is based on 2 measures at 0 to 1 years and 3 measures at 2 to 3 years. In these cases, the natural fluctuation of ability may be more pronounced. We deal with this issue in two ways. We conduct a sensitivity analysis, whereby we exclude the measures of SLCN, which are highly skewed, namely PEDS language measures. We find that the re-estimated coefficients remain consistent (results not reported) and use this re-estimated model to exclude SLCN outliers. It is also important to recognize that the trade-off to ensuring a normal distribution, is that we are losing the natural interindividual variation, which are informative to our results. This approach may limit generalizability of our results. Secondly, as discussed above, the lack of genetic information of our sample may reduce the power of any effects relating to the genetic makeup of infants and their language acquisition.

## 5. Conclusions

In this paper, we present new evidence that improving environmental protective factors can lead to significant expansion in the acquisition of speech, language, and communication skills in infants. The study identified specific aspects of the environment to be leveraged to produce optimal language development. Interventions focused on supporting families to optimize home learning environments and enabling improvements in environmental protective factors, are key and even children with a poor start to life can catch up, but the compounding effects of perinatal care and birth should not be overlooked.

## Figures and Tables

**Table 1:** Descriptive analysis.

Variable	Measurement	*n* = 5089		
		Mean	95% CI	
*Section A. Outcome variables*			−95%	+95%
Speech language and communication needs (SLCN)	z-score (Range −4.7 to 1.54)	−0.04	−0.08	0.00
Speech	z-score (Range −2.1 to 2.1)	−0.10	−0.16	−0.04
Language	z-score (Range −6.5–1.0)	−0.02	−0.05	0.02
Communication	z-score (Range −2.3 to 2.3)	−0.03	−0.10	0.03
*Section B. Family characteristics*				
Socio-economic position	z-score (Range −4.3 to 3.1)	0.28	0.22	0.33
Equivilised income	Annual gross household income equivilised (Range 0 to $4647)	$678	$645	$712
Single parent	Receiver of single parent benefit 1 = Yes, 0 = No	0.54	0.07	1.02
Hardship scale	Range 0 to 6	0.33	0.28	0.38
Stressful life Index	Range 0 to 20	1.19	1.10	1.28
Mother’s depression (reverse coded)	Range 0 to 6	4.48	4.45	4.52
Mother’s Year 12	1 = Yes, 0 = No	77.55	74.85	80.25
Mother’s general health	1 = Excellent,2 = Very Good,3 = Good, 4 = Fair, 5 = Poor	2.27	2.21	2.32
Fathers’ general health	1 = Excellent,2 = Very Good,3 = Good, 4 = Fair, 5 = Poor	2.41	2.36	2.47
Non-English-speaking background	1 = Yes, 0 = No	9.65	7.74	11.56
Indigenous	1 = Yes, 0 = No	1.52	0.73	2.31
Mother’s parenting self-efficacy	Range 1 to 10	8.49	8.42	8.56
Father’s parenting self-efficacy	Range 1 to 10	7.63	7.54	7.73
Mother’s parental warmth	Range 1 to 5	4.51	4.49	4.54
Father’s parental warmth	Range 1 to 5	4.23	4.20	4.26
Mother’s parenting hostility	Range 1 to 10	1.99	1.91	2.07
Father’s parenting hostility	Range 1 to 10	2.07	2.00	2.14
*Section C. Perinatal risk*				
Prescription medication taken through pregnancy	1 = Yes, 0 = No	90.56	88.67	92.45
Average daily alcohol during pregnancy	Number (Range 0 to 2.5)	0.07	0.06	0.08
Foods excluded during pregnancy	1 = Yes, 0 = No	48.05	51.28	44.82
Mother had gestational diabetes	1 = Yes, 0 = No	5.64	4.15	7.13
Mother had high blood pressure during pregnancy	1 = Yes, 0 = No	7.05	5.39	8.71
Other physical issue during pregnancy	1 = Yes, 0 = No	19.85	17.27	22.43
Mother had mental health issues during pregnancy	1 = Yes, 0 = No	14.75	12.46	17.04
Antidepressants medication taken during pregnancy	1 = Yes, 0 = No	2.06	1.14	2.98
Average daily cigarettes during pregnancy	Number (range 0 to 55)	0.71	0.54	0.88
Mothers Age	Age in years (Range 15 to 63)	31.47	31.17	31.78
Number of perinatal medical visits	Number (Range 0 to 6)	5.65	5.61	5.69
*Section D. Birth risk*				
Infant has low body weight	1 ≤ 2500 gm	2.71	3.76	1.66
Infant’s gestational age at birth	Age in weeks (Range 22 to 50)	39.24	39.14	39.35
Infant’s head circumference	z-score (Range −17.2 to 4.4)	−0.23	−0.29	−0.18
Infant’s length	z-score (Range −13.9 to 4.8)	0.34	0.28	0.40
Infant birth weight for length	z-score (Range −6.9 to 4.2)	−0.18	−0.26	−0.11
Infant needed intensive care after birth	1 = Yes, 0 = No	1.86	1.84	1.88
Infant needed ventilator after birth	1 = Yes, 0 = No	1.97	1.96	1.98
Number of days in hospital after birth				
with private hospital insurance	Days (Range 0 to 354)	6.04	5.74	6.35
w/o private hospital insurance	Days (Range 0 to 340)	5.20	5.61	5.69
Single or multiple birth	1 = single, 2 = twin, 3 = triplet	4.77	0.32	0.38
Infant is breast fed	1 = Yes, 0 = No	90.56	88.67	92.45
Age stopped beast feeding	Age (years) (Range o to 1.2)	0.34	0.32	0.36
		***n* = 5089**		
**Variable**	**Measurement**	**Mean**	**95% CI**	
*Section A. Outcome variables*			−95%	+95%
*Section E. Community risk*				
SEIFA economic index	Quintiles 1–5	0.35	0.32	0.38
SEIFA advantage/disadvantage index	Quintiles 1–6	0.28	0.25	0.31
Community earning <1 K per month	Range 0 to 100	51.54	50.63	52.46
Community employment rate	Range 19–94	40.69	39.82	41.56
SEIFA education and occupation	Range 780–1240	1002	997	1007
*Section F: Genetic risk*				
Father has long-term medical condition	1 = Yes, 0 = No	17.03	14.60	14.60
Mother has long-term medical condition	1 = Yes, 0 = No	23.10	20.38	20.38
Multi-generational alcohol problem	1 = Yes, 0 = No	9.73	7.91	11.56
Multi-generational mental health issues	1 = Yes, 0 = No	17.81	15.37	20.26
*Section G: Infant Characteristics*				
Gender	Female = 0, Male = 1	53.90	50.68	57.13
Infants health status				
Excellent	1 = Yes, 0 = No	53.42	0	100
Very good	1 = Yes, 0 = No	28.93	0	100
Good and below	1 = Yes, 0 = No	17.65	0	100
Infant has special health care needs	1 = Yes, 0 = No	5.31	3.86	6.77
Weekly child care attendance	Hours (Range 0 to 72)	9	8	10
Number of older siblings				
0 siblings	1 = Yes, 0 = No	39.33	38.38	40.27
1 sibling	1 = Yes, 0 = No	36.46	33.71	35.56
2 or more siblings	1 = Yes, 0 = No	22.18	25.19	26.89
		***n* = 5089**		
**Variable**	**Measurement**	**Mean**	**Range**	
*Section A. Outcome variables*			*Min*	*Max*
*Section E. Community risk*				
SEIFA economic index	Quintiles 1–5	3.09	1	5
SEIFA advantage/disadvantage index	Quintiles 1–6	3.03	1	5
Community earning <1 K per month	Range 0 to 100	51.51	0	91
Community employment rate	Range 19–94	40.73	13	84
SEIFA education and occupation	Range 780–1240	1003	690	1220
*Section F: Genetic risk*				
Father has long-term medical condition	1 = Yes, 0 = No	17.26	0	100
Mother has long-term medical condition	1 = Yes, 0 = No	23.09	0	100
Multi-generational alcohol problem	1 = Yes, 0 = No	9.74	0	100
Multi-generational mental health issues	1 = Yes, 0 = No	17.88	0	100
*Section G: Infant Characteristics*				
Gender	Female = 0, Male = 1,	53.72	0.00	1.00
Infant health status	1 = Excellent,2 = Very Good, 3 = Good and below	1	1	3
Infant has special health care needs	1 = Yes, 0 = No	5.29	0	100
Weekly childcare attendance	Hours	9	0	72
Number of older siblings	Number	1	0	10

SEIFA = Socio-Economic Indexes for Areas, w/o = without. Characteristics reported in wave 1 when infants are aged birth to 1 years of age.

**Table 2:** SLCN Regression analysis.

Outcome Variable						
			SLCN	Domains		
			(Standardized Score)	Speech	Language	Communication
Col/Row		Explanatory Variable	1	2	3	4
Environmental	1	Family Index ^a^	0.153 **	0.081 **	0.143 **	0.241 **
			(8.76)	(2.98)	(7.73)	(7.25)
	2	Community Index ^b^	−0.017 *	−0.038 **	−0.000	−0.044 **
			(−2.45)	(−3.41)	(−0.06)	(−3.30)
	3	Perinatal Index ^c^	0.055 **	0.021	0.048 **	0.095 **
			(3.29)	(0.77)	(2.98)	(3.05)
	4	Birth Index ^d^	0.074 **	0.092 **	0.052 **	0.100 **
			(5.28)	(4.39)	(3.77)	(4.04)
Gen	5	Genetic Index ^e^	0.004	0.007	−0.006	−0.001
			(0.42)	(0.49)	(−0.59)	(−0.05)
Infant	6	Male	−0.073 **	−0.058 **	−0.059 **	−0.102 **
			(−6.48)	(−3.41)	(−5.28)	(−4.98)
	7	General Health				
		Very good	−0.062 **	-0.051 **	−0.055 **	−0.076 **
			(−5.15)	(−4.46)	(−4.46)	(−3.39)
		Average and below	−0.104 **	−0.111 **	−0.111 **	−0.061 +
			(−5.59)	(−5.56)	(−5.56)	(−1.86)
	8	Special health care needs	−0.048 *	−0.117 **	−0.036	−0.098 *
			(−2.13)	(−1.57)	(−1.57)	(−2.36)
	9	Childcare attendance	−0.001	−0.001	−0.001 *	0.001
			(−1.35)	(−1.38)	(−2.08)	(1.43)
	10	Number older siblings (3 or more)	−0.050 **	−0.066 **	−0.027 **	−0.091 **
			(−7.63)	(−6.76)	(−4.32)	(−7.46)
	11	Age 2 to 3 yrs	−0.050 **	−0.066 **	−0.027 **	−0.091 **
			(−7.63)	(−6.76)	(−4.32)	(−7.46)
		Population mean	0.046 **	0.073 **	0.036 *	0.009
		Observations	(2.77)	(2.69)	(2.14)	(0.28)
		rho	0.185	0.043	0.114	0.216

^a^ Family Index = 17 components: socio-economic position (SEP), equivalized income, single parent, hardship scale, stressful life index, mothers depression, mothers education, mothers health, fathers health, non-English-speaking background, indigenous status, mothers parenting self-efficacy, warmth, and hostility, fathers parenting self-efficacy, warmth, and hostility; ^b^ Community Index = 5 components: SEIFA index of economic resources, SEIFA index of relative socio-economic advantage and disadvantage, Year 12 completion, SEIFA index of education and occupation and employment rates in the area of residence; ^c^ Perinatal Index = 11 components: maternal age (squared, reverse coded), presence of perinatal care (number of medical appts), pregnancy healthy behaviors (smoking and alcohol, reverse coded), presence of mental health issues (yes/no), high blood pressure (yes/no) and gestational diabetes (yes/no); ^d^ Birth Index = 10 components: LBW, gestational age, infants head circumference, infant’s length, infant required intensive care, infant required ventilator, number of days in hospital and single or multiple birth. ^e^ Genetics Index = 10 components: parents long-term health conditions, grandparent’s mental health (yes/no) and grandparents alcohol issues (yes/no). *p*-value < 0.01 **, <0.05 *, <0.10 +.

**Table 3:** Mediating factors of SLCN.

		Mediating Factors (Significant at 5 Percent Level *)							
		SLCN		Domains					
		(Standardised Score)		Speech		Language		Communication	
Row	Explanatory Variable	1		2		3		4	
1	Family Index ^a^	M_self efficacy	0.044 **	NESB	0.070 **	Z_singleparent	−0.033 +	NESB	0.047 **
		Stressful life	0.013 +	M_self efficacy	0.046 **	Stressful life Index	0.016 *	M_self efficacy	0.058 **
		M_warmth	0.068 **	M_warmth	0.064 **	SEP	0.034 **	M_warmth	0.129 **
		F_warmth	0.025 **	F_warmth	0.029 *	M_depression	−0.019 *	F_warmth	0.026 *
				M_hostility	0.022 +	NESB	−0.035 **		
				Hardship scale	0.051 **	M_self efficacy	0.034 **		
						M_warmth	0.042 **		
						F_warmth	0.020 **		
2	Community Index ^b^	SEIFA economic	−0.029 +					SEIFA economic	−0.087 **
								SEIFA adv_dis	0.122 *
							0.004	SEIFA ed/occ	−0.071 +
3	Peri-natal ^c^	High BP	0.011 +	Smoking	−0.038 **	Food excluded	0.012 +	Prescription medication taken	0.026 *
		Smoking	−0.021 **	Mental health issues	−0.006	Prescription medication taken	0.013 *	Alcohol	−0.028 *
		Mother’s Age	−0.025 **	Mother’s Age	−0.049 **			Smoking	−0.029 *
		Prescription medication taken	0.014 *	Alcohol	−0.022 *			High blood pressure	0.035 **
								Mother’s Age	−0.069 **
4	Birth Index ^d^	LBW	−0.071	Somewhat early 33–36 weeks	−0.059 *	LBW	−0.089 +	Somewhat early 33–36 weeks	−0.096 *
		Somewhat early 33–36 weeks	−0.018 *	Single birth	0.020 +			Very early 32 weeks and under	−0.193 *
		Very early 32 weeks and under	−0.179 *					Single birth	0.036 **
		Single birth	0.018 *						
5	Genetic Index ^e^	M_medical condition	0.055 **	Multi-generational risk (father)	−0.047 *	M_medical condition	0.054 **	M_medical condition	0.063 *

M = mother, F = father, SEIFA = Socio-economic Indexes for Areas, SEIFA ed/occ = SEIFA education and occupation, LBW = low birth weight, BMI = body mass index, Gest Age = Gestational Age categories, Single parent = receiver of single parent benefit. ^a^ Family Index = 17 components: socioeconomic position (SEP), Equivalised income, single parent, hardship scale, stressful life index, mothers depression, mothers education, mothers health, fathers health, Non-English speaking background, Indigenous status, mothers parenting self-efficacy, warmth and hostility, fathers parenting self-efficacy, warmth and hostility; ^b^ Community Index = 5 components: SEIFA Index of economic resources, SEIFA index of relative socio-economic advantage and disadvantage, Year 12 completion, SEIFA index of education and occupation and employment rates in the area of residence; ^c^ Peri-natal Index = 11 components: maternal age (squared, reverse coded), presence of peri-natal care (number of medical appts), pregnancy healthy behaviours (smoking and alcohol, reverse coded), presence of mental health issues (yes/no), high blood pressure (yes/no) and gestational diabetes (yes/no); ^d^ Birth Index = 10 components: LBW, gestational age, infants head circumference, infant’s length, infant required intensive care, infant required ventilator, number of days in hospital and single or multiple birth. ^e^ Genetics Index = 10 components: parents long term health conditions, grandparent’s mental health (yes/no) and grandparents alcohol issues (yes/no). *p*-Value < 0.01 **, <0.05 *, <0.10 +.

**Table 4:** Interactions with age of child (time).

Outcome Variable							
Explanatory Variable				SLCN	Domains		
				(Standardized Score)	Speech	Language	Communication
				1	2	3	4
Environmental	1	Family Index ^a^	Age 0 to 1 years	0.137 **	0.141 **	0.108 **	0.214 **
				(5.60)	(3.12)	(4.68)	(4.74)
			Age 2 to 3 years	0.170 **	0.005	0.187 **	0.274 **
				(7.52)	(0.22)	(6.61)	(6.88)
	2	Community Index ^b^	Age 0 to 1 years	−0.039 **	−0.061 **	−0.016*	−0.071 **
				(−4.04)	(−3.27)	(−1.97)	(−3.91)
			Age 2 to 3 years	0.012	−0.007	0.020 +	0.001
				(1.38)	(−0.77)	(1.83)	(0.09)
	3	Perinatal Index ^c^	Age 0 to 1 years	0.049 *	0.047	0.014	0.120 **
				(2.09)	(1.03)	(0.78)	(2.90)
			Age 2 to 3 years	0.065 **	−0.008	0.091 **	0.056
				(3.18)	(−0.34)	(3.49)	(1.44)
	4	Birth Index ^d^	Age 0 to 1 years	0.102 **	0.151 **	0.057 **	0.141 **
				(5.06)	(4.13)	(3.30)	(3.93)
			Age 2 to 3 years	0.038 *	0.027	0.045 *	0.043
				(2.19)	(1.51)	(2.10)	(1.49)
Genetics	5	Genetic Index ^e^	Age 0 to 1 years	−0.015	−0.009	−0.018	−0.012
				(−1.07)	(−0.34)	(−1.46)	(−0.46)
			Age 2 to 3 years	0.028 *	0.021	0.011	0.010
				(2.27)	(1.40)	(0.81)	(0.55)

^a^ Family Index =17 components: socio-economic position (SEP), Equivalized income, single parent, hardship scale, stressful life index, mothers depression, mothers education, mothers health, fathers health, non-English-speaking background, indigenous status, mothers parenting self-efficacy, warmth, and hostility, fathers parenting self-efficacy, warmth, and hostility; ^b^ Community Index = 5 components: SEIFA index of economic resources, SEIFA index of relative socio-economic advantage and disadvantage, Year 12 completion, SEIFA index of education, and occupation and employment rates in the area of residence; ^c^ Perinatal Index = 11 components: maternal age (squared, reverse coded), presence of perinatal care (number of medical appts), pregnancy healthy behaviors (smoking and alcohol, reverse coded), presence of mental health issues (yes/no), high blood pressure (yes/no) and gestational diabetes (yes/no); ^d^ Birth Index = 10 components: LBW, gestational age, infants head circumference, infant’s length, infant required intensive care, infant required ventilator, number of days in hospital and single or multiple birth. ^e^ Genetics Index = 10 components: parents long-term health conditions, grandparent’s mental health (yes/no) and grandparents alcohol issues (yes/no). *p*-Value < 0.01 **, <0.05 *, <0.10 +.

**Table 5:** SLCN algorithm at 2 to 3 years.

		SLCN	Domains		
Row	Average SLCN at 2/3 Years		Speech	Language	Communication
1	0, 0, 0	0.040 **	0.116 **	0.020 +	0.017
2	−1 SD Birth	0.002	0.089 **	−0.025	−0.026
3	+1 SD Birth	0.078 **	0.144 **	0.065 **	0.060 *
4	−1 SD Perinatal	−0.025	0.125 **	−0.071 *	−0.039
5	+1 SD Perinatal	0.105 **	0.108 **	0.111 **	0.073 +
6	−1 SD Family	−0.130 **	0.111 **	−0.167 **	−0.257 **
7	+1 SD Family	0.210 **	0.122 **	0.208 **	0.291 **
8	−1 SD Birth and −1 SD Perinatal	−0.064 *	0.098 **	−0.116 **	−0.081
9	+1 SD Birth and +1 SD Perinatal	0.144 **	0.135 **	0.156 **	0.115 *
10	−1 SD Birth and +1 SD Perinatal	0.067 *	0.081 *	0.066 +	0.030
11	+1 SD Birth and −1 SD Perinatal	0.013	0.152 **	−0.026	0.004
12	−1 SD Birth and −1 SD Family	−0.168 **	0.084 *	−0.212 **	−0.300 **
13	+1 SD Birth and 61:61+1 SD Family	0.248 **	0.149 **	0.252 **	0.334 **
14	−1 SD Birth and +1 SD Family	0.210 **	0.122 **	0.208 **	0.291 **
15	+1 SD Birth and −1 SD Family	−0.091 **	0.138 **	−0.122 **	−0.214 **
16	−1 SD Perinatal and −1 SD Family	−0.195 **	0.119 **	−0.258 **	−0.312 **
17	−1 SD Perinatal+ 1 SD Family	0.275 **	0.114 **	0.298 **	0.346 **
18	+1 SD Perinatal and −1 SD Family	−0.064 +	0.103 **	−0.077 +	−0.201 **
19	−1 SD Perinatal and +1 SD Family	0.183 **	0.157 **	0.162 **	0.278 **
20	−1 SD Perinatal and −1 SD Family and −1 SD Birth	−0.233 **	0.092 *	−0.303 **	−0.355 **
21	+1 SD Perinatal and +1 SD Family and +1 SD Birth	0.313 **	0.141 **	0.343 **	0.389 **
22	−1 SD Perinatal and & −1 SD Family and +1 SD Birth	−0.156 **	0.146 **	−0.213 **	−0.270 **
23	−1 SD Perinatal and +1 SD Family and −1 SD Birth	0.106 **	0.103 **	0.072	0.193 **
24	+1 SD Perinatal and −1 SD Family and −1 SD Birth	−0.103 *	0.076 +	−0.122 *	−0.244 **
25	+1 SD Perinatal and +1 SD Family and −1 SD Birth	0.236 **	0.086 *	0.253 **	0.304 **
26	−1 SD Perinatal and +1 SD Family and +1 SD Birth	0.183 **	0.157 **	0.162 **	0.278 **
27	+1 SD Perinatal and −1 SD Family and +1 SD Birth	−0.026	0.130 **	−0.032	−0.159 *

Family Index =17 components: socio-economic position (SEP), Equivalized income, single parent, hardship scale, stressful life index, mothers depression, mothers education, mothers health, fathers health, non-English-speaking background, indigenous status, mothers parenting self-efficacy, warmth, and hostility, fathers parenting self-efficacy, warmth, and hostility; Community Index = 5 components: Socio-economic Index of economic resources (SEIFA), SEIFA index of relative socio-economic advantage and disadvantage, Year 12 completion, SEIFA index of education, and occupation and employment rates in the area of residence; Perinatal Index = 11 components: maternal age (squared, reverse coded), presence of perinatal care (number of medical appts), pregnancy healthy behaviors (smoking and alcohol, reverse coded), presence of mental health issues (yes/no), high blood pressure (yes/no) and gestational diabetes (yes/no); Birth Index = 10 components: LBW, gestational age, infants head circumference, infant’s length, infant required intensive care, infant required ventilator, number of days in hospital and single or multiple birth. Genetics Index = 10 components: parents long-term health conditions, grandparent’s mental health (yes/no) and grandparents alcohol issues (yes/no). *p*-value < 0.01 **, <0.05 *, <0.1 +.

## Data Availability

The Longitudinal Study of Australian Children (LSAC) dataset is available from the National Centre for Longitudinal Data (NCLD) using Dataverse.

## Appendix A

**Table A1:** Definitions of SLCN.

Speech		
0 to 1 years	Communication and Symbolic Behavior Scales (CSBS) speech composite score [33]	Measures use of sounds and words (out of 17 points).
2 to 3 years	Speech and understanding difficulties	Parent reported measure if child has difficulties speaking, unclear, finding words, putting together, understanding others, unusual voice, stutter/lisp (Yes/No).
2 to 3 years	Speech pathology	Child is receiving speech pathology.
**Language**		
0 to 1 years	CSBS symbolic composite [33],	Measures understanding of words and use of objects (out of 14 points)
0 to 1 years	PEDS receptive and expressive language [30]	Parent reported question is concern about how the child talks and makes speech sounds (Expressive) and how the child understands what the parent says (receptive). (No; a little; Yes).
2 to 3 years	MCDI vocabulary and grammar scales [34].	Number of words.
2 to 3 years	Speech pathology	Child is receiving speech pathology.
**Communication**		
0 to 1 years	CSBS social composite [33]	Measures communicative behaviors emotion and use of eye gaze, communication and gestures (score out of 26).
2 to 3 years	Communications skills scales [30]	Parent reported questionnaire, carries out instructions, asks for question repeated, follows conversation, passes on message, uses speech that can be understood, clearly explains things (Never/sometimes/always).
2 to 3 years	Speech pathology	Child is receiving speech pathology.

SLCN scores were standardized using the transformation of raw scores to Z-scores at each age group.
